# Characterization of New Polymer Material of Amino-β-Cyclodextrin and Sodium Alginate for Environmental Purposes

**DOI:** 10.3390/membranes13040447

**Published:** 2023-04-19

**Authors:** Kinga Kozieł-Trąbska, Sandra Żarska, Tomasz Girek, Wojciech Ciesielski

**Affiliations:** Faculty of Science and Technology, Jan Dlugosz University in Czestochowa, Armii Krajowej Ave. 13/15, 42-201 Czestochowa, Poland

**Keywords:** β-cyclodextrin polymer, composite material, sodium alginate, amino-β-cyclodextrin polymer

## Abstract

The β-cyclodextrin polymer (PβCD) cross-linked with pyromellitic dianhydride (PD) and functionalized with an amino group (PAβCD) was introduced into a matrix made of sodium alginate (SA). Scanning electron microscopy (SEM) images showed a homogeneous surface of the composite material. Infrared spectroscopy (FTIR) testing of the PAβCD confirmed polymer formation. The tested polymer increased its solubility relative to the polymer without the amino group. Thermogravimetric analysis (TGA) confirmed the stability of the system. Differential scanning calorimetry (DSC) showed the chemical binding of PAβCD and SA. Gel permeation chromatography (GPC-SEC) showed high cross-linking of PAβCD and allowed for accurate determination of its weight. The formation of the composite material such as PAβCD introduced into a matrix made of sodium alginate (SA) has several potential environmental implications, including the use of sustainable materials, reduced waste generation, reduced toxicity, and improved solubility.

## 1. Introduction

Hydrogels (HG) are three-dimensional polymer networks capable of absorbing large amounts of water [1]. Hydrogel composites (HGC) are chemically and physically stable, have a smooth, flexible polymer network, and are reusable and multifunctional. These flexible polymer materials retain the ability to swell and retain the right amount of water in their structure, but without dissolving in water [2]. Their main development can be seen in the field of environmental cleaning. Composites based on hydrogels show excellent absorption efficiency in removing inorganic pollutants, including heavy metals, as well as organic pollutants, including drugs and pesticides. One of the main challenges in the modification of hydrogels is to obtain a fully regenerative reusable material, while maintaining its stability.

Sodium alginate (SA) is the sodium salt of alginic acid, obtained from the natural environment, most often from the cell walls of brown algae. It is a white-yellow powder, soluble in water. It thickens in an aqueous solution and forms a gel in the presence of a divalent calcium ion. The main use of this natural polymer is water purification. The physicochemical properties of SA allow for the efficient absorption of heavy metals such as Cu, Cd, and Pb [3,4]. The multitude of functional groups allows the absorption of organic compounds such as drugs [5,6,7], pesticides, and herbicides [8,9,10].

β-cyclodextrin (βCD) is a starch derivative, non-toxic to humans and easily available. The cyclic structure with a visible cavity allows for the formation of inclusion complexes of hydrophobic compounds. Despite the average solubility, it is very popular as a base for systems that absorb hydrophilic compounds. Cross-linked β-cyclodextrin polymers (PβCD) are a group of polymers based on β-cyclodextrins linked with a cross-linking compound [11,12,13]. Particular attention is paid to cross-linked polymers, which have an abundant porous structure, as they are promising absorbent systems. The modification of cyclodextrin hydroxyl groups with substituents significantly affects the interaction between the polymer and the absorbed compounds [14]. Cyclodextrin polymers are successfully used as pollution-absorbing systems [15,16,17].

In the first stage, a modified β-cyclodextrin polymer was obtained. β-cyclodextrin was monosubstituted at position 6 with an amino group. This significantly increased the solubility of the compound. In the next step, the amino derivative was protected with a protecting group (BOC) to reduce cross-linking at this site. Cross-linking was carried out using a bifunctional linker—pyromellitic dianhydride. The synthesis was carried out and described in earlier articles [18,19]. It introduces hydroxyl and carboxyl groups into the system. A polymer system containing both cationic and anionic groups was obtained. Thanks to this, it gained the status of polyampholyte. Modification of the system with amino groups made it possible to obtain a polymer with higher solubility compared with the polymer without modification. Moreover, the porous system contains cavities, not only derived from β-cyclodextrins, but also as spaces in the network. The polymer system was suspended with sodium alginate. Using encapsulation in an electric field, hydrogel composites were created, capable of absorbing inorganic and organic compounds.

The use of β-cyclodextrin as a carrier molecule in polymers of intrinsic microporosity (PIMs) has been extensively studied due to its ability to selectively complex with a variety of guest molecules. The amine-functionalized β-cyclodextrin polymer used in this study may have potential as a carrier molecule in PIMs, particularly in applications related to removing inorganic and organic contaminants [20]. Furthermore, the monodisperse and branched polymer system with a high degree of porosity and better solubility relative to PβCD may also have implications in drug delivery systems, where the polymer can encapsulate and release drugs in a controlled manner [21].

## 2. Materials and Methods

### 2.1. Reagents and Solvents

β-Cyclodextrin (βCD), N, N-dimethyl formamide (DMF), and sodium hydride were purchased from Sigma-Aldrich, St. Louis, MO, USA. DMF was distilled under a vacuum. The dried DMF was stored in a dark bottle over calcium hydride. Sodium hydride (60% in oil) was dried with hexane. Sodium alginate with low viscosity was purchased from Buchi, Switzerland, and pyromellitic dianhydride (PA) was purchased from Alfa Aesar, Ward Hill, MA, USA. Acetone, acetic acid, and hexane were purchased from Chempur, Piekary Slaskie, Poland. Mono-6-azido-6-deoxy-β-cyclodextrin (NβCD), Mono-6-amino-6-deoxy-β-cyclodextrin (AβCD), and blocking the amine group by BOC (BOCAβCD) were synthesized according to the procedure [19].

### 2.2. Synthesis of Polymer BOCAβCD Crosslinked with Pyromellitic Anhydride (PAβCD)

The synthesis was analogously performed per the method described in BOCAβCD (1 g, 1.62 mmol) and was dissolved in DMF (17.59 mL). NaH (0.01 g, 7.5 mmol) was washed with hexane and added in one portion with vigorous. The mixture was stirred for 24 h. After this time, PA (0.73 g, 7.97 mmol) was added in one portion, and the solution was mixed for another 24 h. The product was precipitated with acetone and dried in a vacuum desiccator at room temperature [19].

### 2.3. Preparation of Sodium Alginate/Poly-amino-β-cyclodextrin (SAPAβCD) Microparticles

Microparticles were formed using the vibration technique on a Buchi B-395 Pro encapsulator. The 1.5% solution of alginate and a 0.5% aqueous solution of the resulting polymer (60 mL) were prepared. The parameters of the encapsulation are given in Table 1. An amount of 1 mM CaCl_2_ crosslinker solution was made. A solution of the polymer and alginate fell into the curing solution placed on a magnetic stirrer.

### 2.4. Characterization of the PAβCD

#### 2.4.1. FT-IR Measurement

A Fourier-transform infrared spectroscopy (FT-IR) measurement was prepared on an FT-IR spectrophotometer (Nexus Nicolet, Waltham, MA, USA) in the wavelength range of 4000–400 cm^−1^, with a resolution of 4 cm^−1^ and the number of scans equal to 32. Potassium bromide (KBr) pellets and the test sample were compressed using a manual hydraulic press.

#### 2.4.2. GPC/SEC Analysis

Gel permeation chromatography with size-exclusion chromatography (GPC/SEC) analysis was performed on a 1260 Infinity II Multi-Detector GPC/SEC System equipped with an LS, RI, and viscometry detector by Agilent (Santa Clara, CA, USA). The tests were performed using water with 0.02% NaN_3_ as the eluent and two PLaquagel-OH Mixed-H 300 × 7.5 mm chromatographic columns (Agilent Technologies, Santa Clara, CA, USA) at a flow rate of 1.0 mL/min. A solution of the sample in water at a concentration of 1 mg ml^−1^ was prepared. A 50 µL solution was injected into the chromatographic system. Recorded chromatograms were analyzed by Agilent GPC/SEC Software.

#### 2.4.3. XRD Measurement

The reaction product was analyzed by X-ray powder diffraction (XRD) using a Rigaku MiniFlex 600 powder diffractometer (Curadiation), Rigaku, Tokyo, Japan. The samples were powdered before measurement. The structures were refined by the Rietveld method using the procedures of the FullProf software package.

#### 2.4.4. Solubility Test

The water solubility test was analogously carried out to the method [22]. A saturated solution was made from the tested polymer. The excess of the substance was added to 5 mL of water and stirred for 2 h at 25 °C. The undissolved excess was centrifuged in an MPW-260R centrifuge from MPW Med. Instruments—15,000 rpm and 25 °C, 20 min. An amount of 1 mL of the supernatant solution was taken (ms). The collected solution was evaporated, and the precipitate was weighed (mp). The solubility (S) was obtained following Equation (1).

S(%) = (mp/ms) × 100%,(1)

### 2.5. Characterization of the SAPAβCD

#### 2.5.1. SEM Measurement

A VEGA3 TESCAN instrument (Tescan, Brno, Czech Republic) was used to obtain information on the surface characteristics of the PAβCD. Samples were used in powdery forms. All samples were subjected to a beam energy of 5 kV.

#### 2.5.2. TGA Measurement

Thermogravimetric analysis (TGA) was recorded on a STARe System TGA/DSC 3+ thermal analyzer by Mettler Toledo (Greifensee, Switzerland) calibrated by indium, zinc, and aluminum. A sample of the tested material in open alumina crucibles was placed in the measuring chamber and heated from 0 to 1000 °C with a temperature increase of 20 °C/min, in the range of 0–500 °C inert gas flow at 90 mL/min, and in the range of 500–1000 °C air flow at 90 mL/min. The measurements were duplicated. Recorded thermograms were analyzed with Mettler Toledo STARe Evaluation Software.

#### 2.5.3. DSC Measurement

Differential scanning calorimetry (DSC) was recorded on a STARe System DSC 3 thermal analyzer by Mettler Toledo (Switzerland), calibrated by indium and zinc. A sample of the tested material in an aluminum crucible with a lid with an opening was placed in the measuring chamber, heated from 0 to 500 °C with a temperature increase of 20 °C/min. Inert gas flow was at 90 mL/min. The measurements were duplicated. Recorded thermograms were analyzed with Mettler Toledo STARe Evaluation Software.

## 3. Results and Discussion

### 3.1. Synthesis of Polymer BOCAβCD Crosslinked with Pyromellitic Anhydride (PAβCD)

**Figure 1:**
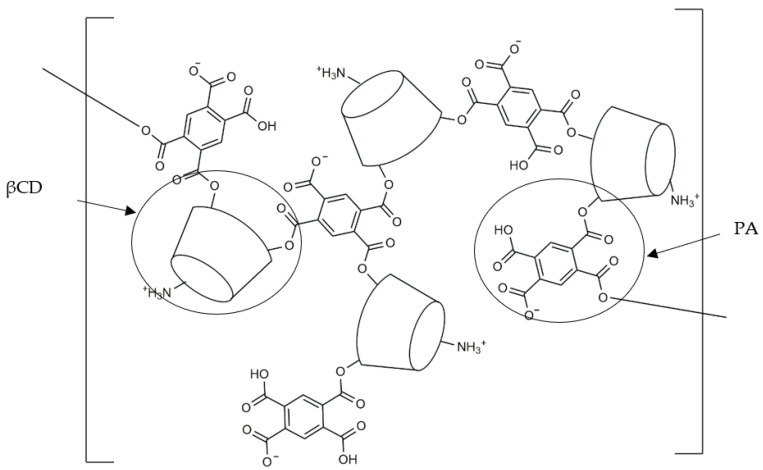
The scheme of polymer structure.

Synthesis was analogously performed to the method described in [18,19]. The β-cyclodextrin enriched with the amine group was used to create the polymer, with significantly improved solvent properties. The well-known pyromellitic dianhydride (PA) was used as the cross-linking agent. NaH was used to activate the hydroxyl groups. The cross-linking reaction was based on the deprotonation of the hydroxyl groups in the β-cyclodextrin ring in the C2 position and the attachment of PA. Deprotonation and crosslinking took place in dry DMF. During cross-linking, the solution turns into a gel which dissolves upon stirring. The obtained polymer contains both anionic and cationic groups as shown in Figure 1.

### 3.2. Preparation of Sodium Alginate/Poly-amino-β-cyclodextrin (SAPAβCD) Microparticles

**Figure 2:**
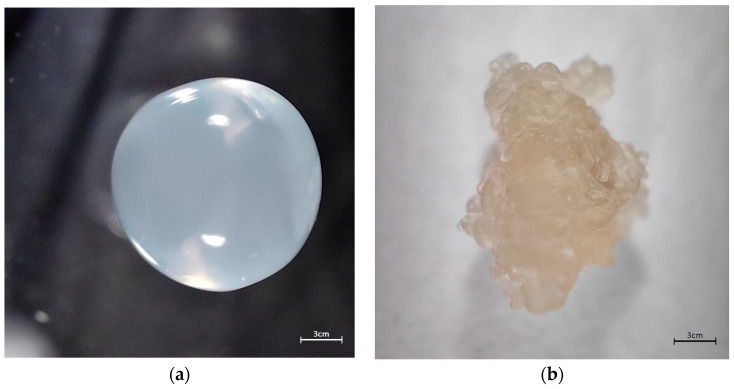
Pictures of microparticles SAPAβCD (**a**) from encapsulator 40× zoom and (**b**) dried 40× zoom.

Using the encapsulator, particles of various sizes between 160 and 2000μm were obtained. The diameter of the particles increases twice the size of the nozzle. The surface is smooth and uncracked. Figure 2 shows the particle magnified 40 times (a) and the particles after drying (b). Particles were air dried at ambient temperature. The weight of the particles is reduced by 95% after drying.

### 3.3. Characterization of the PAβCD

#### 3.3.1. FT-IR Measurement

**Figure 3:**
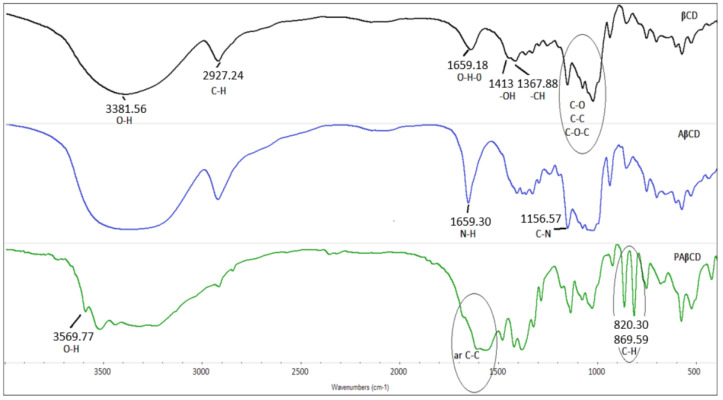
FTIR spectra of βCD, AβCD, and PAβCD.

FTIR spectral analysis was performed to confirm the functional groups. This is possible by providing information in the form of characteristic peaks or changes in their intensity. These spectra are shown in Figure 3. The PAβCD spectrum contains characteristic deformation bands 869 cm^−1^ and 820 cm^−1^ in the dactyloscopic region originating from PA. The spectrum also shows the disappearance of skeletal bands in the region of 2000–1500 cm^−1^ coming from the anhydride ring, due to its opening during cross-linking. In the range of 2200–2000 cm^−1^, there are overtones coming from PA. A strong band O-H 3569.77 cm^−1^ appeared from the carbonyl group formed after the anhydride ring broke. The above bands were compared with the spectrum of AβCD and βCD.

#### 3.3.2. GPC-SEC Analysis

**Figure 4:**
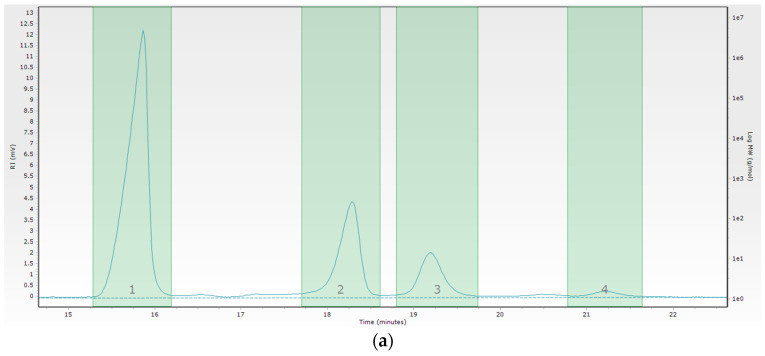

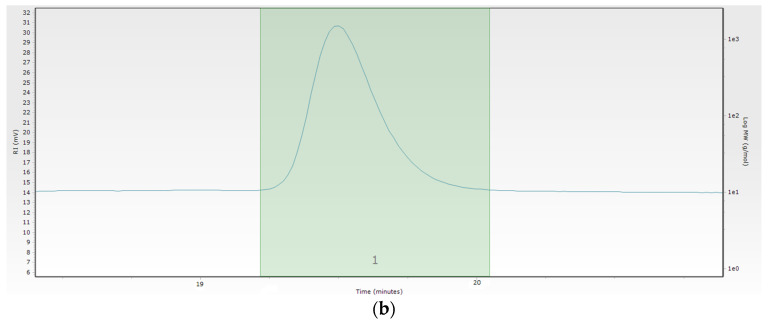
Chromatogram of PAβCD (**a**) and βCD (**b**).

The molecular weight distributions of the PAβCD are shown as GPC-SEC curves in Figure 4a. The retention times of the masses were: 15.95 min for the first fraction, 18.49 min for the second fraction, 19.53 for the third fraction, and 21.41 for the fourth fraction. Peak number 4 is the off-scale peak of the calibration curve. The molecular weight distributions of the βCD are shown as GPC-SEC curves in Figure 4b. The retention time of the mass was 19.53 min. The data collected from the GPC-SEC chromatograms are presented in Table 2.

The weight-average molecular weight (M_w_) for peak 1 was 13.7 kDa, and 5.4 kDa for peak 2. M_w_ for peak 3 was 1.1 kDa. The number-average molecular weight (M_n_) was successively 13.7 kDa and 5.3 Da. M_n_ for peak 3 was 1.1 kDa. The mass distribution values for the 3rd peak, close to the molecular weight of βCD, suggest its presence in the unpolymerized state. The synthesized polymer was characterized by polydispersity close to 1.

**Figure 5:**
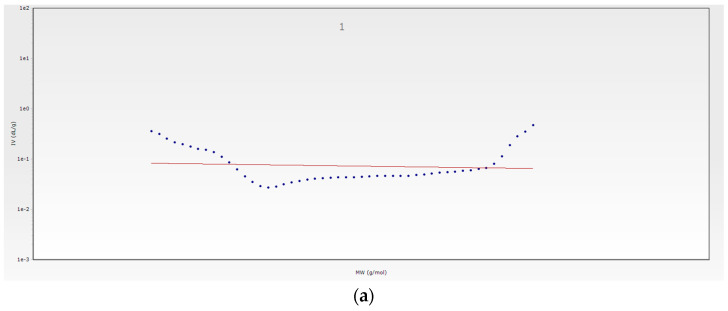

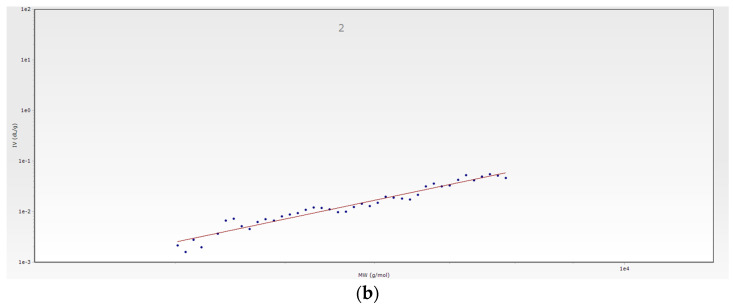
Mark–Houwing plot of PAβCD peak 1 (**a**) and PAβCD peak 2 (**b**).

The branching of individual polymer fractions was marked using Mark–Houwink plots (Figure 5). The slope of the curve for the first PABCD peak (Figure 5a) is slight. The fraction is unbranched. The slope of the curve for the second peak is high (α = 4.68), which indicates its significant branching (Figure 5b).

#### 3.3.3. XRD Measurement

**Figure 6:**
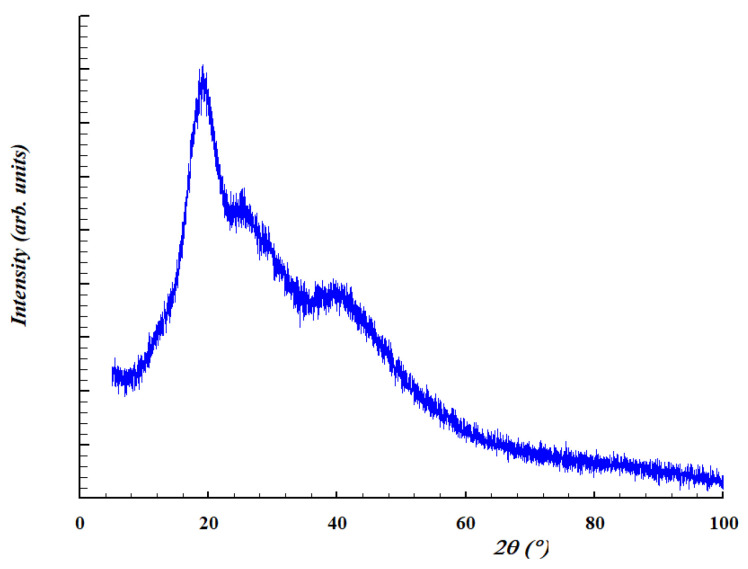
XRD diffractogram of PAβCD.

The XRD diffractogram is shown in Figure 6. The PAβCD diffractogram shows broad, low-intensity bands compared with the sharp and intense βCD peaks reported in the literature [23,24]. This indicates the amorphous nature of the polymer. The crystallinity of βCD and PD decreases, increasing the sites available for bonds.

#### 3.3.4. Solubility Test

**Figure 7:**
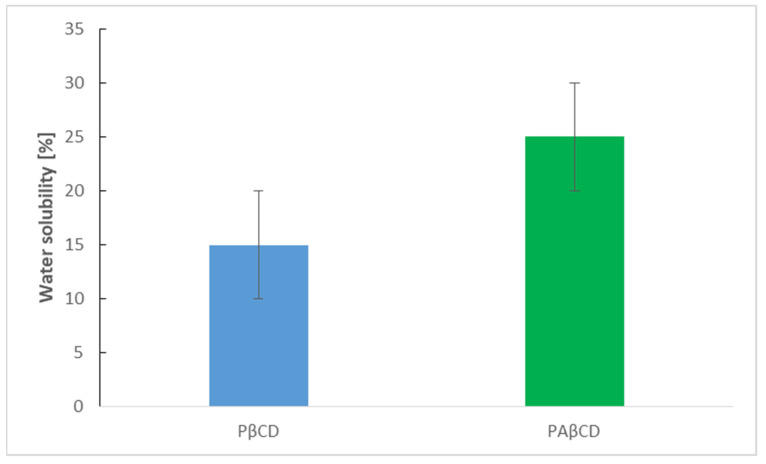
The scheme of polymer solubility.

The solubility of highly cross-linked b-cyclodextrin polymers is an important parameter affecting their functionality. As shown in Figure 7, the value of WS (%) for PAβCD increased by about 10% compared with PβCD. This can be attributed to the amino groups present in PAβCD.

### 3.4. Characterization of the SAPAβCD

#### 3.4.1. SEM Measurement

**Figure 8:**
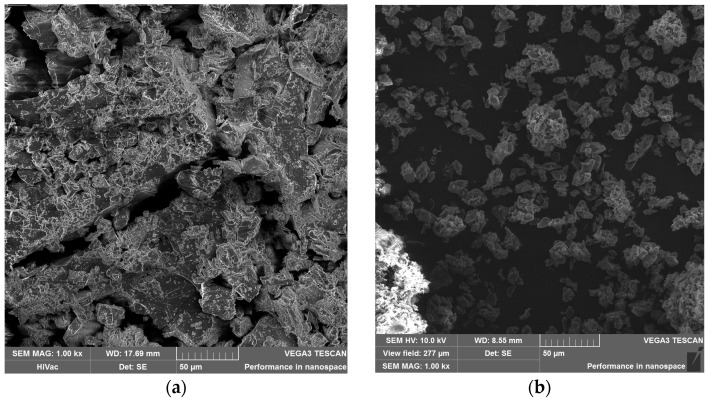

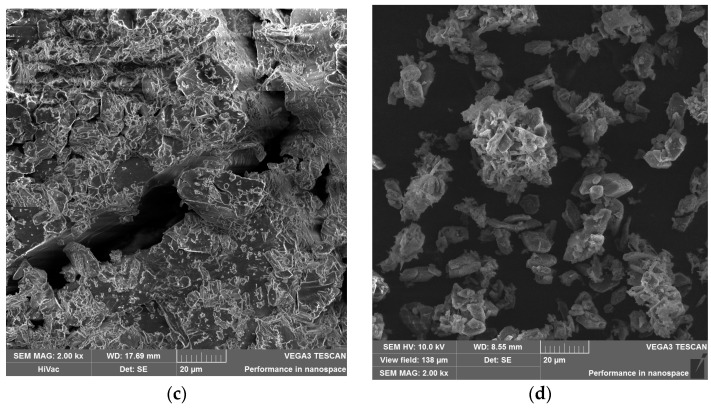
SEM images of βCD at (**a**) 1000× zoom and (**b**) 2000× zoom, and PaβCD at (**c**) 1000× zoom and (**d**) 2000× zoom.

As shown in Figure 8a,c βCD exists as amorphous irregular crystals [25], while PAβCD (Figure 8b,d) is observed as sharp-edged crystals. Image comparison shows that PAβCD is structurally different from βCDs, also in terms of size (βCD particles are up to 200 μm in size, while PAβCD particles are around 20 μm).

#### 3.4.2. TGA Measurement

**Figure 9:**
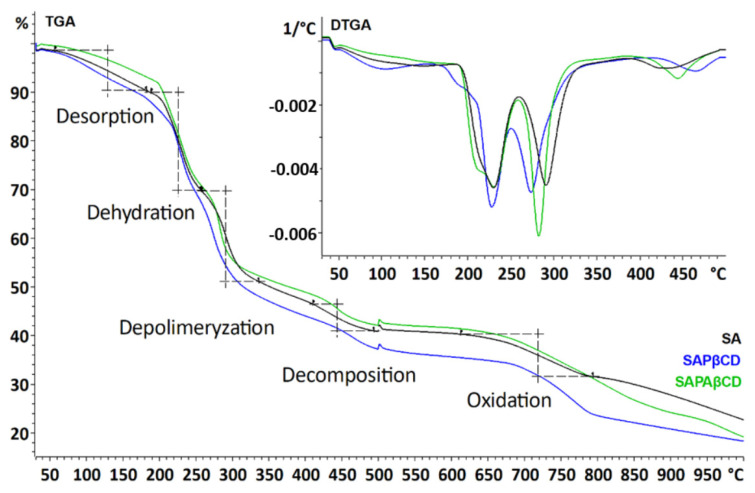
TGA and DTGA thermogram of SA, SAPβCD, and SAPAβCD.

The TGA thermogram of SA shows five weight losses (Figure 9). The first is in the range of 49–181 °C, which is related to dehydration at the level of 8% [26]. The second and third losses are between 183 and 336 °C and are associated with depolymerization, including loss of volatile components, chain breakage, and fragmentation of alginate—approx. 39%. The final decomposition of alginate occurred in the range of 391–500 °C, with a value of approx. 6% and its oxidation is between 627 and 812 °C (loss of approx. 9%).

The thermogram for SAPβCD is similar to that of sodium alginate. It also shows five weight losses. The first is in the range of 49–154 °C, related to dehydration at the level of 10%. The second and third losses are between 201 and 333 °C at the level of approx. 37%—depolymerization. The final decomposition occurred in a similar temperature range (428–500 °C), with a value of approx. 6% and its oxidation is between 624 and 847 °C (loss of approx. 10%).

The same situation occurred for SAPAβCD. The thermogram consists of five mass losses. The first is in the range of 51–154 °C (dehydration at the level of approx. 8%). The second and third losses are between 180 and 329 °C at the level of approx. 38%—depolymerization. The final decomposition took place in a similar temperature range (394–500 °C), with a value of approx. 6% and its oxidation is between 627 and 812 °C (loss of approx. 10%). Such similar thermograms of each of the compounds confirm the chemical bonding between the polymers. Detailed results of the thermal analysis are presented in Table 3.

**Figure 10:**
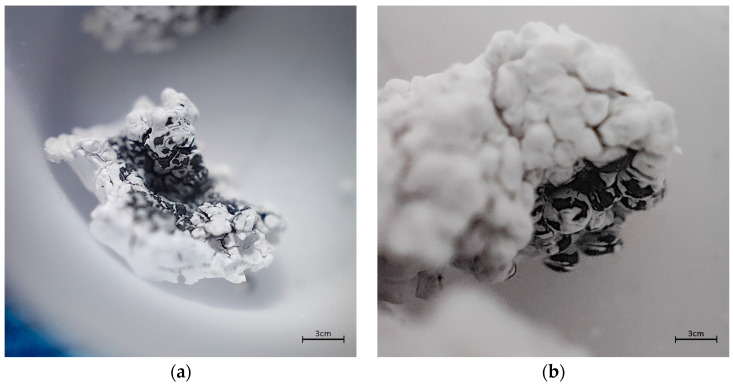
Imagines of SAPAβCD after TGA analysis: (**a**) 20× zoom; (**b**) 40× zoom.

Macrospheres during the last stage of TG measurement were charred and covered with sodium oxide (Figure 10). The emitted gases include water, carbon (II) and (IV) oxides, nitrogen oxides, and hydrides, which may come from amino groups in the polymer structure.

#### 3.4.3. DSC Measurement

**Figure 11:**
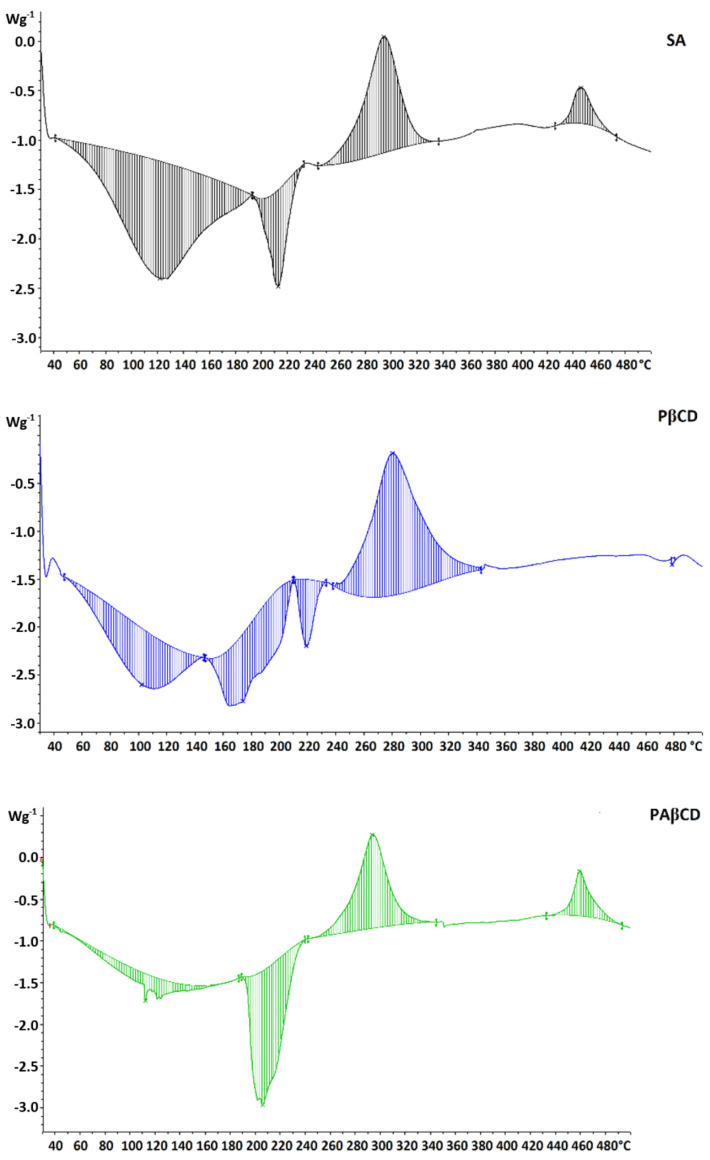
DSC thermograms of SA, SAPβCD, and SAPAβCD.

DSC thermogram SA contains four thermal effects (Figure 11). The first with a peak at 121.03 °C is an exothermic effect of −251.34 J/g, which is related to dehydration. Another is the exothermic effect of −47.6 J/g, associated with the initial stage of depolymerization (partitioning of the polymer chain)—peak 212.14 °C. The third effect is a large endothermic peak of 11.82 J/g at 294.02 °C, corresponding to further depolymerization. The last endothermic peak at 455.77 °C corresponds to the decomposition of SA (−18.69 J/g).

The thermogram for SAPβCD contains five thermal effects. Four of them correspond to effects for SA. The first, with a peak at 101.81 °C, is an exothermic effect of −91.66 J/g, which is related to dehydration. It is less energetic, which indicates a weaker binding of water in the structure of the system. The second peak that did not appear in the thermogram for SA was the depolymerization peak of PβCD (−90.38 J/g, 173.28 °C). The next two are SA depolymerization with the value of −22.37 J/g and 185.53 J/g at the temperatures of 218.69 and 280.27 °C, respectively. The last peak is the decomposition of the whole structure (−478.09 J/g).

A slightly different thermogram can be presented for SAPAβCD. The thermogram contains four thermal effects. The first of them is low in energy, but strongly stretched, with a value of −32.70 J/g, which is associated with dehydration. The change in the appearance of the peak resulted from a different method of drying the compound. Another exothermic effect is the combined effect of depolymerization of PβCD and SA. This is suggested by its energy value of −124.29 J/g. The last two are endothermic effects from SA depolymerization and thermal decomposition of the entire structure (100.29 J/g and 27.59 J/g) at temperatures of 293.75 and 459.27, respectively.

## 4. Conclusions

In this study, a composite material consisting of sodium alginate and an amine-functionalized β-cyclodextrin polymer was successfully formed. On the basis of the results obtained for PAβCD, it can be concluded that a monodisperse and branched polymer system with the same length of particle chains was obtained. This polymer is amorphous compared with native βCD, with better solubility relative to PβCD. The surface of the polymer shows significant porosity with a large number of fissures. The resulting material has a large surface area, high porosity, and excellent adsorption properties. It can selectively adsorb and remove various contaminants from water, such as heavy metals, organic pollutants, and dyes. Furthermore, highly cross-linked cyclodextrin polymers have been utilized as catalysts for various reactions, such as oxidation, reduction, and esterification. The large surface area and porosity of the polymer provide a large number of active sites for catalysis, while the hydrophobic interior of the cyclodextrin cavity can selectively accommodate certain substrates. The SAPAβCD composite material is a thermally stable system with no significant deviations in thermogravimetric analysis relative to SA. The surface of SAPAβCD is homogeneous and smooth, without cracks or scratches.

These discoveries have the effect of expanding the range of SAPAβCD practical applications. The presented polymer system can be a good material for various pharmaceutical and medical applications.

In addition, the obtained SAPAβCD composite material can be used in many environmental applications such as removing inorganic and organic contaminants, potentially providing an eco-friendly solution to environmental pollution. The potential use of the composite material in pharmaceutical, biomedical, and environmental applications could reduce waste generation by providing a material that is durable and can be reused multiple times. The better solubility of the composite material compared with PβCD could reduce the number of solvents needed during production and the environmental impact of solvent disposal. The use of sodium alginate as a matrix material is a sustainable choice because it is derived from natural sources such as brown seaweed.

In summary, highly cross-linked cyclodextrin polymers have shown great potential as versatile materials with a wide range of applications, from environmental remediation to drug delivery and catalysis.

## Figures and Tables

**Table 1:** The parameters of the encapsulation.

Nozzle Diameter (µm)	Concentration of Alginate (%)	Flow Rate (mL/min)	Frequency (Hz)	Voltage (V)	Amplitude
1000	2	30	100	500	0.4
450	1.5	9	300	500	0.5
80	1	1	2000	500	0.6

**Table 2:** The parameters of the GPC-SEC analysis.

**PAβCD**					
**Peak No.**	**Max. RT (min)**	**M_p_ (kDa)**	**M_n_ (kDa)**	**M_w_ (kDa)**	PD *
1	15.95	13.7	13.7	13.7	1.000
2	18.49	5.0	5.3	5.4	1.021
3	19.53	1.1	1.1	1.1	1003
**βCD**					
**Peak No.**	**Max. RT (min)**	**M_p_ (kDa)**	**M_n_ (kDa)**	**M_w_ (kDa)**	PD *
1	19.53	1.1	1.1	1.1	1.002

* Polydispersity, PD = M_w_/M_n._

**Table 3:** The parameters of the TGA and DSC analysis.

**TGA Thermogram**				
**SA**				
**Step 1 (%)**	**Step 2 (%)**	**Step 3 (%)**	**Step 4 (%)**	**Step 5 (%)**
8.25	20.16	18.68	5.52	8.72
**Type of effect**				
Dehydration	Depolymerization	Depolymerization	Decomposition	Oxidation
**Temperature limits (°C)**				
49.79–181.58	183.00–258.55	260.25–335.35	391.60–503.64	627.55–812.39
**SAPβCD**				
**Step 1 (%)**	**Step 2 (%)**	**Step 3 (%)**	**Step 4 (%)**	**Step 5 (%)**
10.02	17.09	18.99	6.35	13.00
**Type of effect**				
Dehydration	Depolymerization	Depolymerization	Decomposition	Oxidation
**Temperature limits (°C)**				
46.07–154.31	201.57–251.83	252.34–332.76	428.75–506.61	624.98–847.54
**SAPAβCD**				
**Step 1 (%)**	**Step 2 (%)**	**Step 3 (%)**	**Step 4 (%)**	**Step 5 (%)**
7.73	21.80	16.51	6.33	10.36
**Type of effect**				
Dehydration	Depolymerization	Depolymerization	Decomposition	Oxidation
**Temperature limits (°C)**				
51.50–194.96	180.32–258.97	255.291–329.25	394.70–504.42	627.55–812.39
**DSC thermogram**				
**SA**				
**Effect 1 (J/g)**	**Effect 2 (J/g)**	**Effect 3 (J/g)**	**Effect 4 (J/g)**	**Effect 5 (J/g)**
−251.34	-	−47.16	111.82	18.69
**Type of effect**				
Exothermic	-	Exothermic	Endothermic	Endothermic
dehydration	-	depolymerization	depolymerization	decomposition
**Peak (°C)**				
121.03	-	212.14	294.02	455.77
**SAPβCD**				
**Effect 1 (J/g)**	**Effect 2 (J/g)**	**Effect 3 (J/g)**	**Effect 4 (J/g)**	**Effect 5 (J/g)**
−91.66	−90.38	−22.37	185.53	-
**Type of effect**				
Exothermic	Exothermic	Exothermic	Endothermic	-
dehydration	depolymerization	depolymerization	depolymerization	Decomposition
**Peak (°C)**				
101.81	173.28	218.69	280.27	478.09
**SAPAβCD**				
**Effect 1 (J/g)**	**Effect 2 (J/g)**	**Effect 3 (J/g)**	**Effect 4 (J/g)**	**Effect 5 (J/g)**
−32.70	-	−124.29	100.29	27.59
**Type of effect**				
Exothermic	-	Exothermic	Endothermic	Endothermic
dehydration	-	depolymerization	depolymerization	decomposition
**Peak (°C)**				
111.99	-	205.20	293.75	459.27

## Data Availability

Data is contained within the article.
